# 2-Benz­yloxy-1,2,4-triazolo[1,5-*a*]quinazolin-5(4*H*)-one

**DOI:** 10.1107/S1600536811024962

**Published:** 2011-06-30

**Authors:** Rashad Al-Salahi, Geffken Detlef, Bari Ahmed

**Affiliations:** a Department of Pharmaceutical Chemistry, College of Pharmacy, King Saud University, Riyadh 11451, Saudi Arabia; b Department of Chemistry, Institute of Pharmacy, University of Hamburg, Bundesstrasse 45, 20146 Hamburg, Germany

## Abstract

The title compound, C_16_H_12_N_4_O_2_, is a functionalized triazoloquinazoline with a substituted benz­yloxy group attached at the 2-position of a triazole spacer. The triazoloquinazoline fused-ring system is approximately planar (r.m.s. deviation = 0.016 Å) while the benzyl substituent is perpendicular to the ring system, making a dihedral angle of 65.29 (6)°. The phenyl ring of the benz­yloxy moiety is equally disordered over two sets of sites. A centrosymmetric N—H⋯N hydrogen bond connects mol­ecules into dimers.

## Related literature

For the biological activity of related compounds, see: Francis *et al.* (1991 ▶, 1998) ▶; Kim *et al.* (1998 ▶); Geffken *et al.* (2008) ▶. For related structures, see: Al-Salahi (2009 ▶); Al-Salahi & Geffken (2010 ▶); Berezank *et al.* (2008*a* ▶,*b* ▶); Ongini *et al.* (2001 ▶). 
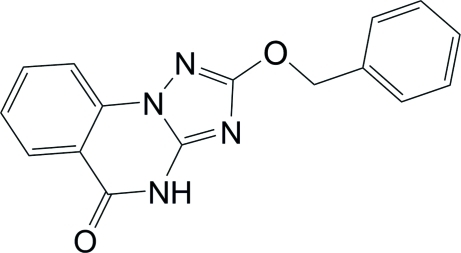


## Experimental

### 

#### Crystal data

C_16_H_12_N_4_O_2_
*M* *_r_* = 292.30
Monoclinic, 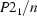

*a* = 5.0319 (15) Å
*b* = 28.207 (9) Å
*c* = 9.408 (3) Å
β = 99.503 (5)°
*V* = 1317.0 (7) Å^3^
*Z* = 4
Mo *K*α radiation
μ = 0.10 mm^−1^
*T* = 153 K
0.50 × 0.10 × 0.03 mm

#### Data collection

Bruker SMART APEX CCD area-detector diffractometer
Absorption correction: multi-scan (*SADABS*; Bruker, 1998 ▶) *T*_min_ = 0.951, *T*_max_ = 0.997
8172 measured reflections
2849 independent reflections
1679 reflections with *I* > 2σ(*I*)
*R*_int_ = 0.053

#### Refinement

*R*[*F* ^2^ > 2σ(*F* ^2^)] = 0.047
*wR*(*F* ^2^) = 0.098
*S* = 0.80
2849 reflections
236 parameters
6 restraints
H-atom parameters constrained
Δρ_max_ = 0.20 e Å^−3^
Δρ_min_ = −0.20 e Å^−3^

### 

Data collection: *SMART* (Bruker, 1998 ▶); cell refinement: *SAINT* (Bruker, 1998 ▶); data reduction: *SAINT*; program(s) used to solve structure: *SHELXS97* (Sheldrick, 2008 ▶); program(s) used to refine structure: *SHELXL97* (Sheldrick, 2008 ▶); molecular graphics: *SHELXTL* (Sheldrick, 2008 ▶); software used to prepare material for publication: *SHELXL97*.

## Supplementary Material

Crystal structure: contains datablock(s) I, global. DOI: 10.1107/S1600536811024962/kp2309sup1.cif

Structure factors: contains datablock(s) I. DOI: 10.1107/S1600536811024962/kp2309Isup2.hkl

Supplementary material file. DOI: 10.1107/S1600536811024962/kp2309Isup3.cml

Additional supplementary materials:  crystallographic information; 3D view; checkCIF report

## Figures and Tables

**Table 1:** Hydrogen-bond geometry (Å, °)

*D*—H⋯*A*	*D*—H	H⋯*A*	*D*⋯*A*	*D*—H⋯*A*
N1—H1⋯N4^i^	0.88	2.19	3.058 (2)	169

Symmetry code: (i) 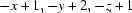.

**Fig. 1.**
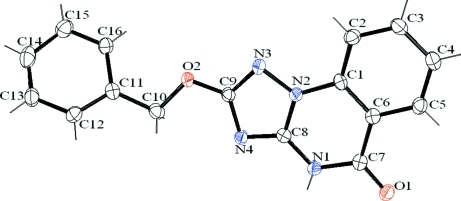
: The molecular structure of the title compound showing 50% probability displacement ellipsoids for non-H atoms and the atom-numbering scheme.

## supplementary crystallographic information

### Comment

Heterocycles with 1,2,4-triazoloquinazoline moiety have been shown to exhibit diverse biological activities. For example, the novel 2-(furan-2-yl)-[1,2,4]triazolo[1,5-*c*]quinazolin-5yl-amine is effective adenosine antagonist (Kim, *et al.*, 1998) whereas the related compound 2-(4-fluoro-phenyl)-[1,2,4]triazolo[1,5-*c*]quinazolin-5-one was found to be benzodiazepine receptor antagonist (Francis, *et al.*, 1988, 1991). In the continuation of our reesearch on triazoloquinazolines, we report herein the results of our study of cyclocondensation of dialkyl-*N*-cyanoimidocarbonates with hydrazinobenzoic acid. 2-alkoxy(arylkoxy)-[1,2,4]triazolo[1,5-*a*]quinazolin-5-ones is an excellent agent for controlling the plant growth diseases caused by fungal agents (Geffken, *et al.*, 2008). The title compound, C_16_H_12_N_4_O_2_, (Fig. 1), consists of quinazoline (C1—C7, C16) and triazole (C8, N2—N4) rings substituted by the benzyl group (C9—C15). The phenyl ring of benzyloxy moiety is disodered over two locations where disordered atoms are in population 0.5:0.5. In the crystal structure, a pairs of intermolecular N—H···.N hydrogen bonds form centrosymmetric dimers (Table 1).

### Experimental

2-Hydrazinobenzoic acid (10 mmol) was added in portion to a stirred solution of dibenzyl-*N*-cyanoimidocarbonate (10 mmol) in ethanol (20 mL) at 273 K. Afterwards triethylamine (30 mmol) was added dropwise over a period of 30 min. After the addition was completed, the reaction mixture was left to stirr overnight at room temperature. Acidification of the mixture was performed by conc. HCl under ice cooling followed by refluxing for 1–2 h. After cooling, the mixture was poured into ice/water, the resulting solid was filtered, washed with water and dried. Recrystallisation from tetrahydrofuran (THF) yielded 2-benzyloxy-4*H*-[1,2,4]triazolo[1,5-*a*]quinazolin-5-one as colourless crystals.

### Refinement

Data corrected for absorption using *SADABS* (Bruker, 1998) and structure solved by direct methods. All nonhydrogen atoms refined as anisotropic by Fourier full matrix least squares. All H atoms were positioned geometrically and refined using a riding model, with C–H = 0.93–0.99 Å. The displacement parameters are *U*_iso_(H) = *U*_eq_(C) where *x* = 1.2 or 1.5. The phenyl ring of benzyloxy moiety is disordered over the two sites with population 0.5 ofatoms in each orientation.

### Crystal data

**Table:**

C_16_H_12_N_4_O_2_	*Z* = 4
*M**_r_* = 292.30	*F*(000) = 608
Monoclinic, *P*2_1_/*n*	*D*_x_ = 1.474 Mg m^−^^3^
*a* = 5.0319 (15) Å	Mo *K*α radiation, λ = 0.71073 Å
*b* = 28.207 (9) Å	µ = 0.10 mm^−^^1^
*c* = 9.408 (3) Å	*T* = 153 K
β = 99.503 (5)°	Needle, colourless
*V* = 1317.0 (7) Å^3^	0.50 × 0.10 × 0.03 mm

### Data collection

**Table:**

Bruker SMART APEX CCD area-detector diffractometer	2849 independent reflections
Radiation source: fine-focus sealed tube	1679 reflections with *I* > 2σ(*I*)
graphite	*R*_int_ = 0.053
ω scans	θ_max_ = 27.0°, θ_min_ = 2.9°
Absorption correction: multi-scan (*SADABS*; Bruker, 1998)	*h* = −6→6
*T*_min_ = 0.951, *T*_max_ = 0.997	*k* = −25→35
8172 measured reflections	*l* = −12→11

### Refinement

**Table:**

Refinement on *F*^2^	Primary atom site location: structure-invariant direct methods
Least-squares matrix: full	Secondary atom site location: difference Fourier map
*R*[*F*^2^ > 2σ(*F*^2^)] = 0.047	Hydrogen site location: inferred from neighbouring sites
*wR*(*F*^2^) = 0.098	H-atom parameters constrained
*S* = 0.80	*w* = 1/[σ^2^(*F*_o_^2^) + (0.0347*P*)^2^ + 0.4968*P*] where *P* = (*F*_o_^2^ + 2*F*_c_^2^)/3
2849 reflections	(Δ/σ)_max_ = 0.005
236 parameters	Δρ_max_ = 0.20 e Å^−^^3^
6 restraints	Δρ_min_ = −0.20 e Å^−^^3^

### Special details

**Table:**

Geometry. All e.s.d.'s (except the e.s.d. in the dihedral angle between two l.s. planes) are estimated using the full covariance matrix. The cell e.s.d.'s are taken into account individually in the estimation of e.s.d.'s in distances, angles and torsion angles; correlations between e.s.d.'s in cell parameters are only used when they are defined by crystal symmetry. An approximate (isotropic) treatment of cell e.s.d.'s is used for estimating e.s.d.'s involving l.s. planes.
Refinement. Refinement of *F*^2^ against ALL reflections. The weighted *R*-factor *wR* and goodness of fit *S* are based on *F*^2^, conventional *R*-factors *R* are based on *F*, with *F* set to zero for negative *F*^2^. The threshold expression of *F*^2^ > σ(*F*^2^) is used only for calculating *R*-factors(gt) *etc*. and is not relevant to the choice of reflections for refinement. *R*-factors based on *F*^2^ are statistically about twice as large as those based on *F*, and *R*- factors based on ALL data will be even larger.

### Fractional atomic coordinates and isotropic or equivalent isotropic displacement parameters (Å^2^)

**Table:**

	*x*	*y*	*z*	*U*_iso_*/*U*_eq_	Occ. (<1)
O1	0.8828 (3)	1.12152 (5)	0.45128 (14)	0.0270 (4)	
O2	0.8556 (3)	0.91207 (5)	0.83235 (14)	0.0245 (4)	
N1	0.7916 (3)	1.04986 (6)	0.54486 (16)	0.0209 (4)	
H1	0.6460	1.0465	0.4796	0.025*	
N2	1.0822 (3)	1.01884 (6)	0.74430 (16)	0.0194 (4)	
N3	1.1110 (3)	0.97886 (6)	0.83206 (17)	0.0231 (4)	
N4	0.7304 (3)	0.97406 (6)	0.65836 (16)	0.0197 (4)	
C1	1.2541 (4)	1.05801 (7)	0.7576 (2)	0.0195 (5)	
C2	1.4809 (4)	1.06091 (7)	0.8647 (2)	0.0237 (5)	
H2	1.5256	1.0359	0.9321	0.028*	
C3	1.6386 (4)	1.10113 (7)	0.8700 (2)	0.0276 (5)	
H3	1.7925	1.1039	0.9430	0.033*	
C4	1.5772 (4)	1.13772 (8)	0.7708 (2)	0.0287 (5)	
H4	1.6891	1.1650	0.7762	0.034*	
C5	1.3524 (4)	1.13430 (7)	0.6640 (2)	0.0236 (5)	
H5	1.3105	1.1592	0.5962	0.028*	
C6	1.1870 (4)	1.09411 (7)	0.6560 (2)	0.0196 (5)	
C7	0.9465 (4)	1.09086 (7)	0.5424 (2)	0.0203 (5)	
C8	0.8573 (4)	1.01485 (7)	0.64452 (19)	0.0180 (4)	
C9	0.8959 (4)	0.95440 (7)	0.7751 (2)	0.0193 (5)	
C10	0.6230 (4)	0.88574 (7)	0.7619 (2)	0.0256 (5)	
H10A	0.6238	0.8843	0.6568	0.031*	
H10B	0.4553	0.9017	0.7779	0.031*	
C11	0.6348 (4)	0.83661 (7)	0.8236 (2)	0.0225 (5)	0.496 (2)
C12	0.5451 (8)	0.79726 (15)	0.7427 (5)	0.0272 (11)	0.496 (2)
H12	0.4805	0.8018	0.6429	0.033*	0.496 (2)
C13	0.5427 (9)	0.75163 (15)	0.7968 (5)	0.0300 (12)	0.496 (2)
H13	0.4760	0.7261	0.7352	0.036*	0.496 (2)
C14	0.6311 (5)	0.74409 (8)	0.9305 (3)	0.0357 (6)	0.496 (2)
H14	0.6246	0.7126	0.9658	0.043*	0.496 (2)
C15	0.7393 (9)	0.78049 (16)	1.0299 (5)	0.0313 (12)	0.496 (2)
H15	0.8071	0.7737	1.1281	0.038*	0.496 (2)
C16	0.7399 (8)	0.82635 (15)	0.9751 (4)	0.0228 (10)	0.496 (2)
H16	0.8099	0.8515	1.0373	0.027*	0.496 (2)
C11'	0.6348 (4)	0.83661 (7)	0.8236 (2)	0.0225 (5)	0.504 (2)
C12'	0.3903 (8)	0.81776 (14)	0.8448 (4)	0.0242 (11)	0.504 (2)
H12'	0.2290	0.8358	0.8244	0.029*	0.504 (2)
C13'	0.3880 (9)	0.77136 (15)	0.8972 (4)	0.0281 (11)	0.504 (2)
H13'	0.2219	0.7576	0.9111	0.034*	0.504 (2)
C14'	0.6311 (5)	0.74409 (8)	0.9305 (3)	0.0357 (6)	0.504 (2)
H14'	0.6357	0.7137	0.9745	0.043*	0.504 (2)
C15'	0.8587 (10)	0.76532 (16)	0.8936 (5)	0.0408 (14)	0.504 (2)
H15'	1.0208	0.7474	0.9042	0.049*	0.504 (2)
C16'	0.8615 (9)	0.81179 (16)	0.8417 (5)	0.0357 (13)	0.504 (2)
H16'	1.0229	0.8253	0.8198	0.043*	0.504 (2)

### Atomic displacement parameters (Å^2^)

**Table:**

	*U*^11^	*U*^22^	*U*^33^	*U*^12^	*U*^13^	*U*^23^
O1	0.0252 (8)	0.0249 (9)	0.0296 (8)	−0.0033 (7)	0.0001 (6)	0.0086 (7)
O2	0.0239 (8)	0.0186 (8)	0.0285 (8)	−0.0063 (7)	−0.0031 (6)	0.0045 (6)
N1	0.0183 (9)	0.0213 (10)	0.0217 (9)	−0.0006 (8)	−0.0007 (7)	0.0038 (7)
N2	0.0187 (9)	0.0168 (9)	0.0218 (9)	0.0002 (8)	0.0001 (7)	0.0019 (7)
N3	0.0256 (10)	0.0168 (9)	0.0259 (9)	−0.0018 (8)	0.0013 (7)	0.0034 (8)
N4	0.0186 (9)	0.0174 (9)	0.0224 (9)	−0.0001 (8)	0.0017 (7)	0.0013 (7)
C1	0.0188 (11)	0.0164 (11)	0.0240 (10)	−0.0016 (9)	0.0056 (8)	−0.0040 (9)
C2	0.0236 (12)	0.0235 (12)	0.0234 (11)	0.0021 (10)	0.0019 (9)	0.0018 (9)
C3	0.0264 (13)	0.0249 (12)	0.0287 (11)	−0.0038 (10)	−0.0034 (9)	−0.0035 (10)
C4	0.0268 (13)	0.0239 (12)	0.0341 (12)	−0.0074 (10)	0.0009 (10)	−0.0001 (10)
C5	0.0278 (12)	0.0174 (11)	0.0258 (11)	0.0013 (10)	0.0050 (9)	0.0007 (9)
C6	0.0189 (11)	0.0188 (11)	0.0217 (10)	0.0031 (9)	0.0055 (8)	−0.0017 (9)
C7	0.0193 (11)	0.0190 (12)	0.0237 (11)	0.0020 (9)	0.0071 (8)	0.0000 (9)
C8	0.0168 (11)	0.0185 (11)	0.0194 (10)	0.0022 (9)	0.0050 (8)	−0.0022 (9)
C9	0.0187 (11)	0.0168 (11)	0.0221 (10)	0.0006 (9)	0.0022 (8)	−0.0003 (9)
C10	0.0206 (12)	0.0254 (13)	0.0296 (11)	−0.0021 (10)	0.0007 (9)	−0.0018 (9)
C11	0.0184 (12)	0.0220 (12)	0.0280 (11)	−0.0004 (10)	0.0071 (8)	−0.0021 (9)
C12	0.027 (3)	0.023 (3)	0.030 (2)	0.003 (2)	−0.0003 (19)	0.0025 (19)
C13	0.027 (3)	0.020 (3)	0.041 (3)	−0.002 (2)	0.001 (2)	0.000 (2)
C14	0.0316 (15)	0.0226 (13)	0.0535 (16)	−0.0040 (11)	0.0088 (12)	0.0055 (11)
C15	0.036 (3)	0.029 (3)	0.029 (2)	0.001 (2)	0.007 (2)	0.006 (2)
C16	0.022 (2)	0.022 (2)	0.025 (2)	−0.0017 (19)	0.0085 (18)	0.0003 (18)
C11'	0.0184 (12)	0.0220 (12)	0.0280 (11)	−0.0004 (10)	0.0071 (8)	−0.0021 (9)
C12'	0.019 (2)	0.023 (3)	0.031 (2)	−0.0005 (19)	0.0057 (18)	−0.0009 (19)
C13'	0.030 (3)	0.026 (3)	0.029 (2)	−0.005 (2)	0.0084 (19)	0.002 (2)
C14'	0.0316 (15)	0.0226 (13)	0.0535 (16)	−0.0040 (11)	0.0088 (12)	0.0055 (11)
C15'	0.028 (3)	0.027 (3)	0.066 (3)	0.003 (2)	0.002 (2)	0.008 (2)
C16'	0.020 (3)	0.028 (3)	0.059 (3)	0.001 (2)	0.008 (2)	0.011 (2)

### Geometric parameters (Å, °)

**Table:**

O1—C7	1.223 (2)	C6—C7	1.479 (3)
O2—C9	1.339 (2)	C10—C11	1.500 (3)
O2—C10	1.450 (2)	C10—H10A	0.9900
N1—C8	1.364 (2)	C10—H10B	0.9900
N1—C7	1.397 (2)	C11—C12	1.379 (5)
N1—H1	0.8800	C11—C16	1.465 (4)
N2—C8	1.351 (2)	C12—C13	1.385 (6)
N2—N3	1.391 (2)	C12—H12	0.9500
N2—C1	1.396 (2)	C13—C14	1.281 (5)
N3—C9	1.320 (2)	C13—H13	0.9500
N4—C8	1.333 (2)	C14—C15	1.435 (5)
N4—C9	1.380 (2)	C14—H14	0.9500
C1—C2	1.394 (3)	C15—C16	1.393 (6)
C1—C6	1.399 (3)	C15—H15	0.9500
C2—C3	1.381 (3)	C16—H16	0.9500
C2—H2	0.9500	C12'—C13'	1.399 (6)
C3—C4	1.392 (3)	C12'—H12'	0.9500
C3—H3	0.9500	C13'—H13'	0.9500
C4—C5	1.387 (3)	C15'—C16'	1.400 (6)
C4—H4	0.9500	C15'—H15'	0.9500
C5—C6	1.401 (3)	C16'—H16'	0.9500
C5—H5	0.9500		
			
C9—O2—C10	115.94 (15)	N2—C8—N1	119.84 (18)
C8—N1—C7	122.56 (16)	N3—C9—O2	118.13 (17)
C8—N1—H1	118.7	N3—C9—N4	117.35 (18)
C7—N1—H1	118.7	O2—C9—N4	124.51 (17)
C8—N2—N3	109.78 (16)	O2—C10—C11	108.60 (16)
C8—N2—C1	124.32 (16)	O2—C10—H10A	110.0
N3—N2—C1	125.89 (16)	C11—C10—H10A	110.0
C9—N3—N2	100.65 (15)	O2—C10—H10B	110.0
C8—N4—C9	100.89 (16)	C11—C10—H10B	110.0
C2—C1—N2	122.16 (18)	H10A—C10—H10B	108.3
C2—C1—C6	121.80 (19)	C12—C11—C16	114.3 (3)
N2—C1—C6	116.03 (17)	C12—C11—C10	122.8 (2)
C3—C2—C1	118.12 (19)	C16—C11—C10	122.9 (2)
C3—C2—H2	120.9	C11—C12—C13	124.5 (4)
C1—C2—H2	120.9	C11—C12—H12	117.7
C2—C3—C4	121.51 (19)	C13—C12—H12	117.7
C2—C3—H3	119.2	C14—C13—C12	119.5 (4)
C4—C3—H3	119.2	C14—C13—H13	120.2
C5—C4—C3	119.9 (2)	C12—C13—H13	120.2
C5—C4—H4	120.1	C13—C14—C15	123.7 (3)
C3—C4—H4	120.1	C13—C14—H14	118.2
C4—C5—C6	120.09 (19)	C15—C14—H14	118.2
C4—C5—H5	120.0	C16—C15—C14	116.6 (4)
C6—C5—H5	120.0	C16—C15—H15	121.7
C1—C6—C5	118.57 (18)	C14—C15—H15	121.7
C1—C6—C7	121.65 (18)	C15—C16—C11	121.4 (4)
C5—C6—C7	119.78 (18)	C15—C16—H16	119.3
O1—C7—N1	120.98 (18)	C11—C16—H16	119.3
O1—C7—C6	123.46 (19)	C13'—C12'—H12'	121.0
N1—C7—C6	115.55 (17)	C12'—C13'—H13'	119.3
N4—C8—N2	111.34 (16)	C16'—C15'—H15'	118.4
N4—C8—N1	128.80 (17)	C15'—C16'—H16'	120.6

### Hydrogen-bond geometry (Å, °)

**Table:**

*D*—H···*A*	*D*—H	H···*A*	*D*···*A*	*D*—H···*A*
N1—H1···N4^i^	0.88	2.19	3.058 (2)	169

Symmetry codes: (i) −*x*+1, −*y*+2, −*z*+1.
