# 2-Benz­yloxy-1,2,4-triazolo[1,5-*a*]quinazolin-5(4*H*)-one. Corrigendum

**DOI:** 10.1107/S1600536811037780

**Published:** 2011-09-17

**Authors:** Rashad Al-Salahi, Detlef Geffken, Bari Ahmed

**Affiliations:** a Department of Pharmaceutical Chemistry, College of Pharmacy, King Saud University, Riyadh 11451, Saudi Arabia; b Department of Chemistry, Institute of Pharmacy, University of Hamburg, Bundesstrasse 45, 20146 Hamburg, Germany

## Abstract

The name of one of the authors in the paper by Al-Salahi *et al.* [*Acta Cryst.* (2011), E**67**, o1861] is corrected.

## Abstract 2

Corrigendum to *Acta Cryst.* (2011), E**67**, o1861.

In the paper by Al-Salahi *et al.* (2011) ▶, the name of the second author is given incorrectly. The correct name is given above.
